# Health perceptions of adverse weather in older adults in England: analysis of 2019/20 survey data

**DOI:** 10.1093/eurpub/ckae153

**Published:** 2024-10-24

**Authors:** Grace Anne Turner, Agostinho Moreira de Sousa, Emer O’Connell, Sari Kovats, Katya Brooks, Owen Landeg, Sharif Ismail, Anusha Rajamani, Shakoor Hajat

**Affiliations:** NIHR Health Protection Research Unit in Environmental Change and Health, Department of Public Health, Environment and Society, London School of Hygiene and Tropical Medicine, London, United Kingdom; NIHR Health Protection Research Unit in Environmental Change and Health, Department of Public Health, Environment and Society, London School of Hygiene and Tropical Medicine, London, United Kingdom; Extreme Events and Health Protection Team, UK Health Security Agency, London, United Kingdom; NIHR Health Protection Research Unit in Environmental Change and Health, Department of Public Health, Environment and Society, London School of Hygiene and Tropical Medicine, London, United Kingdom; NIHR Health Protection Research Unit in Environmental Change and Health, Department of Public Health, Environment and Society, London School of Hygiene and Tropical Medicine, London, United Kingdom; NIHR Health Protection Research Unit in Environmental Change and Health, Department of Public Health, Environment and Society, London School of Hygiene and Tropical Medicine, London, United Kingdom; Extreme Events and Health Protection Team, UK Health Security Agency, London, United Kingdom; NIHR Health Protection Research Unit in Environmental Change and Health, Department of Public Health, Environment and Society, London School of Hygiene and Tropical Medicine, London, United Kingdom; Extreme Events and Health Protection Team, UK Health Security Agency, London, United Kingdom; NIHR Health Protection Research Unit in Environmental Change and Health, Department of Public Health, Environment and Society, London School of Hygiene and Tropical Medicine, London, United Kingdom; Extreme Events and Health Protection Team, UK Health Security Agency, London, United Kingdom; NIHR Health Protection Research Unit in Environmental Change and Health, Department of Public Health, Environment and Society, London School of Hygiene and Tropical Medicine, London, United Kingdom; Extreme Events and Health Protection Team, UK Health Security Agency, London, United Kingdom; NIHR Health Protection Research Unit in Environmental Change and Health, Department of Public Health, Environment and Society, London School of Hygiene and Tropical Medicine, London, United Kingdom

## Abstract

Risks to older adults (OA) (aged 65+ years) associated with hot and cold weather in the UK are well-documented. The study aim is to explore OA perception of health risks from high and low temperatures, health-protective measures undertaken, and implications for public health messaging. In 2019/20, Ipsos MORI conducted face-to-face surveys with OA in England (*n* = 461 cold weather survey, *n* = 452 hot weather survey). Participants reported temperature-related symptoms, risk perceptions for different groups, and behaviours during hot and cold weather. Analysis involved binomial logistic regression models to assess potential factors (demographics, vulnerability, behaviours) associated with older adults’ health risk perception in hot and cold weather. Less than half of OA in both surveys agreed that hot or cold weather posed a risk to their health. OA with higher education, annual income >£25 000 or home ownership were less likely to perceive their health at risk during cold weather and regional differences in hot weather were identified. OA who recognized those the same age or living alone as at an increased risk were more likely to perceive their own health as at risk. OA were more likely to self-identify health risks when reporting those aged 65 yrs+ to be at an increased risk in cold weather. Various temperature-related protective behaviours were associated with older adults’ risk perception in hot and cold weather. These findings provide evidence for public health agencies to target high risk individuals, and modify temperature-related public health messaging to protect OA.

## Introduction

Risks to health from hot and cold weather are well-documented [1, 2]. In 2022, the UK recorded its hottest day at 40.3°C, leading to the first Level 4 Heat Health Alert and an estimated 2985 deaths in England across five heat episodes—the highest annual toll [3]. Additionally, from 2000 to 2019, England and Wales experienced an average of 60 500 excess cold-related deaths annually [4]. UK evidence projects a decrease in cold spells and frost days but anticipates an increase in frequency and intensity of heatwaves [5], however, cold risks will remain significant to the end of the century [6–8]. In 2019–20, there were 28 300 excess winter deaths (excluding Covid-19) in England and Wales, a 20% rise from the previous year. During the summer of 2019, England faced two Level 3 Heat Health Alerts, resulting in an estimated 572 excess deaths among those aged 65 and older during the second heatwave (21st–28th July) [9]. Analysis from 2000 to 2019 showed an average of 800 heat-related deaths and 60 500 deaths linked to cold weather in England and Wales [4]. Exposure to heat and cold contributes to significant excess mortality, with notable differences across regions and population sub-groups. Existing scientific evidence shows that the health impacts from cold and heat are more severe in older adults (OA) (aged 65 years and above) [8, 10]. For instance, the relative risk of death and hospital admission for OA rises during extreme temperatures [11–13]. Additionally, the highest health burden often occurs at moderate temperatures, typically between and 8°C for cold [14, 15]. Cold exposure in OA is linked to increased risks of strokes, cardiovascular diseases, and injuries, while heat exposure may worsen existing respiratory and cardiovascular issues [16]. Whilst, there is concern that heat exposure amongst OA may exacerbate existing respiratory and cardiovascular conditions [16]. Previous literature has indicated that the health risk to OA from changes in temperature are due to impaired thermoregulation, pre-existing health conditions and impaired ability to perform self-protective behaviours [17–20]. Evidence shows that OA often underutilize coping mechanisms during extreme weather due, in part, to a limited perception of health risks [21]. However, factors influencing OA’s perception of risk during temperature extremes remain unclear, potentially affecting their adoption of health-protective behaviours.

A scoping review of OA revealed various social and individual factors that drive health risk perception (HRP) in hot and cold weather. These include limited knowledge of temperature-related health risks, the presence of comorbidities, perceived severity of adverse weather, coping mechanisms, and an external locus of control [5]. Age, income, sex, and living conditions also affect poor HRP [21]. For OA, risk perception in extreme weather relates to self-identification of age and recognition as ‘vulnerable’ [22]. Evidence indicates that social structures and cohesion impact HRPs; e.g. living alone increases both exposure and perceived risk [21]. Additionally, specific vulnerability assessments for cold and heat extremes have shown that financial status, health conditions, literacy, and physical assets like housing insulation significantly influence vulnerability and HRPs among OA [20].

Previous evidence has suggested a weak link between the adoption of temperature-protective behaviours predicting personal HRP in hot or cold weather [5, 23]. However, experience with heat-related health symptoms has been strongly associated with reporting protective behaviours, with a weaker behavioural response during heat exposure [23]. A survey in England reported most OA do undertake some heat protection measures that they consider ‘common sense’ during heatwaves but it is not clear how behavioural responses predict HRP [22]. OA and other vulnerable groups compared to the general population have demonstrated variable uptake of recommended behaviours, e.g. drinking fluids, and opening or closing windows during hot weather, staying active, and dressing warmly in cold weather [8, 24, 25]. Conclusions from previous research have referenced age-related differences in uptake of recommended home and personal protection measures with OA as likely due to limited perceptions of themselves as at risk or vulnerable to extreme heat and cold [18].

The UK Health Security Agency’s report on the Health Effects of Climate Change emphasizes the need for further research on temperature risks to OA [8]. Literature indicates that OA, as a vulnerable group, often do not perceive an increased health risk from hot or cold weather. A scoping review concluded that knowledge of temperature-related health risks does not necessarily enhance risk perception. Factors influencing risk perception include age identity, past experiences, and comorbidities, which can undermine effective risk communication strategies. It is crucial to examine how engaging in protective behaviour influences OA HRP to inform public health decisions and targeted messaging.

Risk communication is vital for heat and cold preparedness; however, challenges in promoting and prioritizing OA HRP may limit effectiveness [26]. Targeted actions and behaviour changes should align with national public health messaging and support policies like the UK Adverse Weather and Health Plan [27]. This article analyses demographic, personal, and behavioural factors affecting OA risk perception from extreme temperatures in England. The representative sample allows for a thorough examination of effective strategies to mitigate health risks associated with temperature extremes. Findings will guide updates to the UKHSA’s Adverse Weather and Health Plan, launched in April 2023, which focuses on behaviour change techniques and the communication of necessary actions [27].

## Methods

### Survey and study participants

We analysed data collected from two surveys conducted by Ipsos MORI that affect heat and cold HRP in OA (aged 65 years and older) in England, commissioned by UKHSA. The heat survey (*n* = 1706) was conducted from 26 July to 4 August 2019 following very hot weather in the UK and Europe and the cold survey (*n* = 1719) took place between 7 and 23 February 2020 following a mild winter (average temperature 5.1°C). February 2020 was characterized as a particularly wet and mild month, even being recorded as the wettest February on record [28]. The risk framing approach adopted to develop the questions presented in each survey was presenting heat or cold as a hazard that can influence the health of the respondents. Both surveys were regionally comparable and conducted across England in person by an Ipsos researcher. The survey sample was nationally representative aged 15 and over. Quotas were set for age by gender, region, working status, and housing tenure. This article presents the results only for OA.

Participants were asked questions relating to their perceptions, awareness, and experience of the risks of recent hot or cold weather events. Initially in both surveys, participants personal risk perception was captured by asking them to respond on a Likert scale to what extent they agreed with the following statement ‘hot/cold weather is a risk to my health’. The surveys cover three themes: participant temperature-related symptoms; participant HRP of other groups (e.g. the very young); and acute participant responses or behaviours to hot and cold weather event (Supplementary material S1). Questions were a mixture of closed yes/no, Likert scale, and listed options for selection. Temperature-related symptom outcomes were not included in the regression analysis but are described in Supplementary material S2.

### Analysis

IBM SPSS (Version 28.0.0.0) was used to re-code and categorize data. The analysis was conducted in R (Version 1.3.1093). First, descriptive and exploratory analyses were carried out (cross tabulations and bivariate correlation matrix) to identify variable relationships. Models were selected from running ordinary least squares regression models (OLSR) with all potentially relevant predictor variables to identify which best predict OA perception of risk to their health from hot/cold weather. OLSR models which reported the lowest akaike information criterion (AIC) value and highest adjusted *R*-squared value were selected.

Binomial logistic regression models were fit to assess potential factors associated with OA HRP in hot or cold weather. The dependent variable for each analysis was comparing agreement (strongly agree/agree) that ‘hot/cold weather is a risk to my health’ (1) versus disagreeing (strongly disagree/agree) or being neutral (neither agree nor disagree) in response to ‘hot/cold weather is a risk to my health’ (0).

Three analyses were carried out for each survey:

- Analysis 1: demographic determinants of OA HRP of hot/cold weather.
- Analysis 2: OA perception of other groups health risk in hot/cold weather as a predictor of own personal risk.
- Analysis 3: OA responses or adopted behaviours association with their HRP in hot/cold weather.

Each analysis adjusted for the following covariates: age, sex, education, annual income, home ownership, and region (South versus Midlands/North). Ethnicity was not included as a covariate due to a small sample size of non-white British.

### Ethics

The data collection process was carried out by Ipsos MORI, a market research company, as the project was reviewed by the internal IPSOS MORI Ethics group (full ethics statement in Supplementary material S3).

## Results

Table 1 describes the characteristics of the sub-sample of OA in each survey [heat survey *n* = 452 (26%), cold survey *n* = 461 (27%)]. Just under half of OA in both surveys agreed with the statement ‘hot/cold weather is a risk to my health’ (47% heat survey, 45% cold survey). Full summary tables of analyses 1–3 in Supplementary material S4.

**Table 1.** Demographic information of survey participants^a^

Variable	Heat survey participants, *n* (%)	Cold survey participants, *n* (%)	General pop. distribution
All survey participants (*n*)	1706	1719	–
Older adults (*n*)	452 (26%)	461 (27%)	18.3%
Age	Mean = 74	Mean = 74	Mean = 73
65–74	241 (14%)	274 (16%)	9.8%
75–84	170 (9%)	135 (7%)	6.1%
85+	41 (2%)	52 (3%)	2.4%
Sex			
Male	219 (48%)	257 (56%)	46%
Female	233 (52%)	204 (44%)	54%
Education			
No formal education	107 (24%)	91 (20%)	18.2%
GCSE/O-level/A-level/vocational qualification	173 (38%)	188 (40%)	30.3%
Higher education (degree or above)	121 (27%)	146 (31%)	33.8%
Other	51 (11%)	36 (8%)	8.1%
Annual income			
0–£24 999	146 (32%)	184 (40%)	–
£25 000–£49 999	85 (19%)	87 (19%)	–
£50 000+	38 (8%)	33 (7%)	–
Unknown or refused	183 (41%)	157 (34%)	–
Ethnicity			
White British	423 (94%)	440 (95%)	90%
Non-White British	29 (6%)	19 (5%)	10%
Employment status			
Employed	43 (9%)	41 (8%)	10%
Retired	409 (91%)	420 (91%)	90%
Home ownership			
Home owned accommodation	380 (84%)	368 (80%)	77%
Rented accommodation	72 (16%)	85 (20%)	23%
Region			
North East	21 (5%)	22 (5%)	5%
North West	68 (15%)	61 (13%)	13%
Yorkshire & the Humber	47 (10%)	39 (8%)	10%
East Midlands	38 (8%)	52 (11%)	9%
West Midlands	41 (9%)	39 (8%)	11%
East	68 (15%)	59 (13%)	12%
London	62 (14%)	44 (10%)	10%
South East	61 (14%)	68 (15%)	17%
South West	46 (10%)	77 (17%)	12%

a Older Adults (aged 65 years and over).

### Demographic determinants of older adult HRP

Regional differences in OA HRP dependent on hot or cold weather across England are observed between the surveys (Supplementary material S5). Across the Midlands, East of England and Yorkshire & Humber, a higher percentage of respondents perceived their health to be at risk in hot weather than cold weather, ranging from 47% to 63% compared to 29% to 52% respectively. In the North West, 64% of OA in the cold weather survey agreed their health was at risk during cold weather compared to 46% of OA in the hot weather survey.

**Figure 1.**
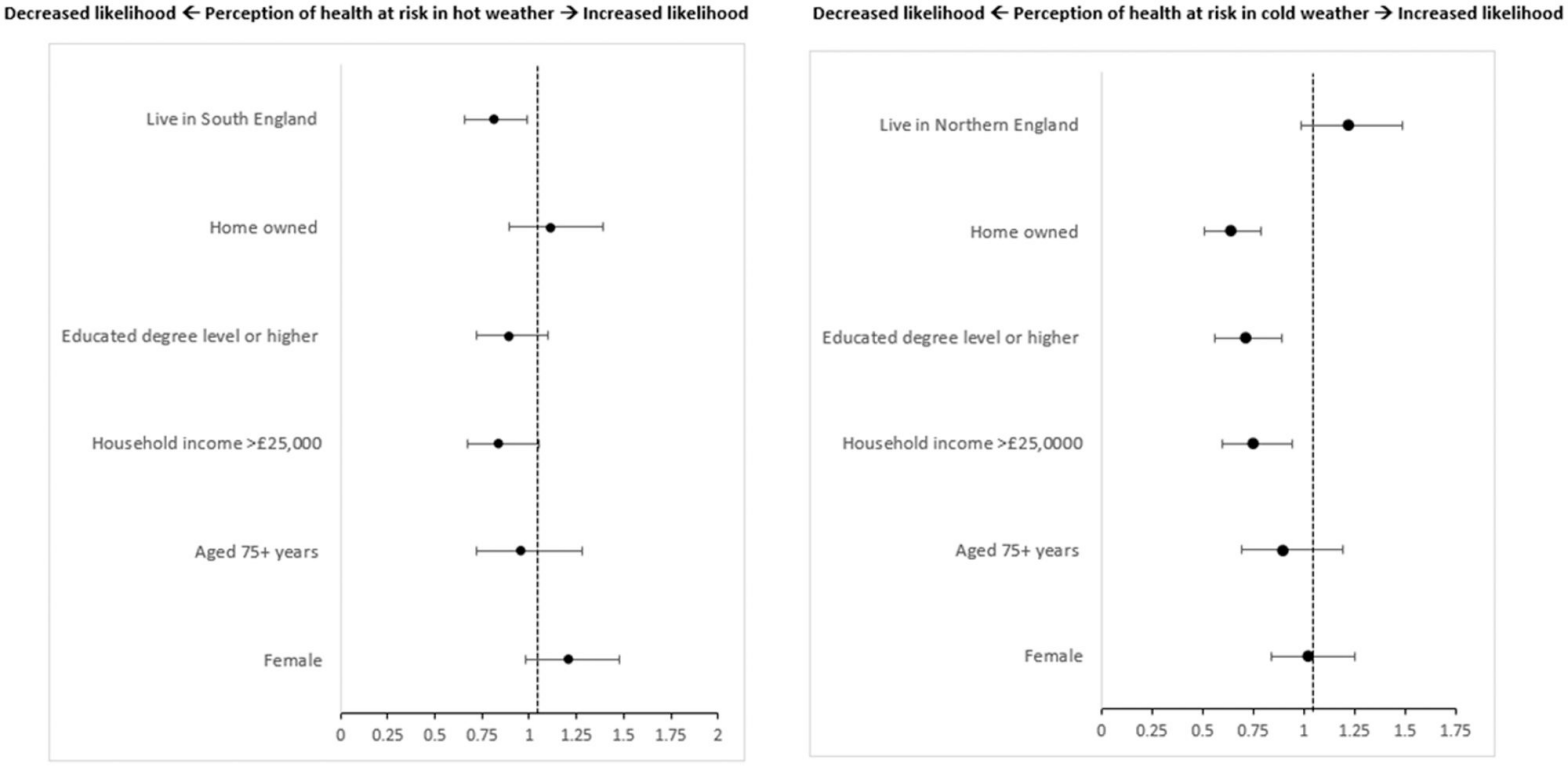
Analysis 1—binomial logistic regression model plot showing demographic predictors of OA (aged 65+ years) perception of risk to health from hot (left) and cold (right) weather. Figure shows odds ratios. Adjusted for age, sex, education, income, home tenancy, and region.

OA who had a degree or higher [odds ratio, OR 0.71, 95% confidence interval, CI (0.56–0.89)], household income over £25 000 a year [OR 0.75, 95% CI (0.60–0.94)], or owned their own home [OR 0.64, 95% CI (0.51–0.79)] were significantly less likely to perceive their health as at risk in cold weather (Fig. 1). OA who lived in the South of England (South West, South East, or London) were 19% less likely to perceive their health to be at risk during hot weather compared to those living in the Midlands or Northern England [OR 0.81, 95% CI (0.66–0.99)], however the association is weak (*P* = .043). No other determinant (such as age or income) predicted OA perceiving themselves at risk from high temperatures.

### Perception of other OA risk in hot and cold weather

**Figure 2.**
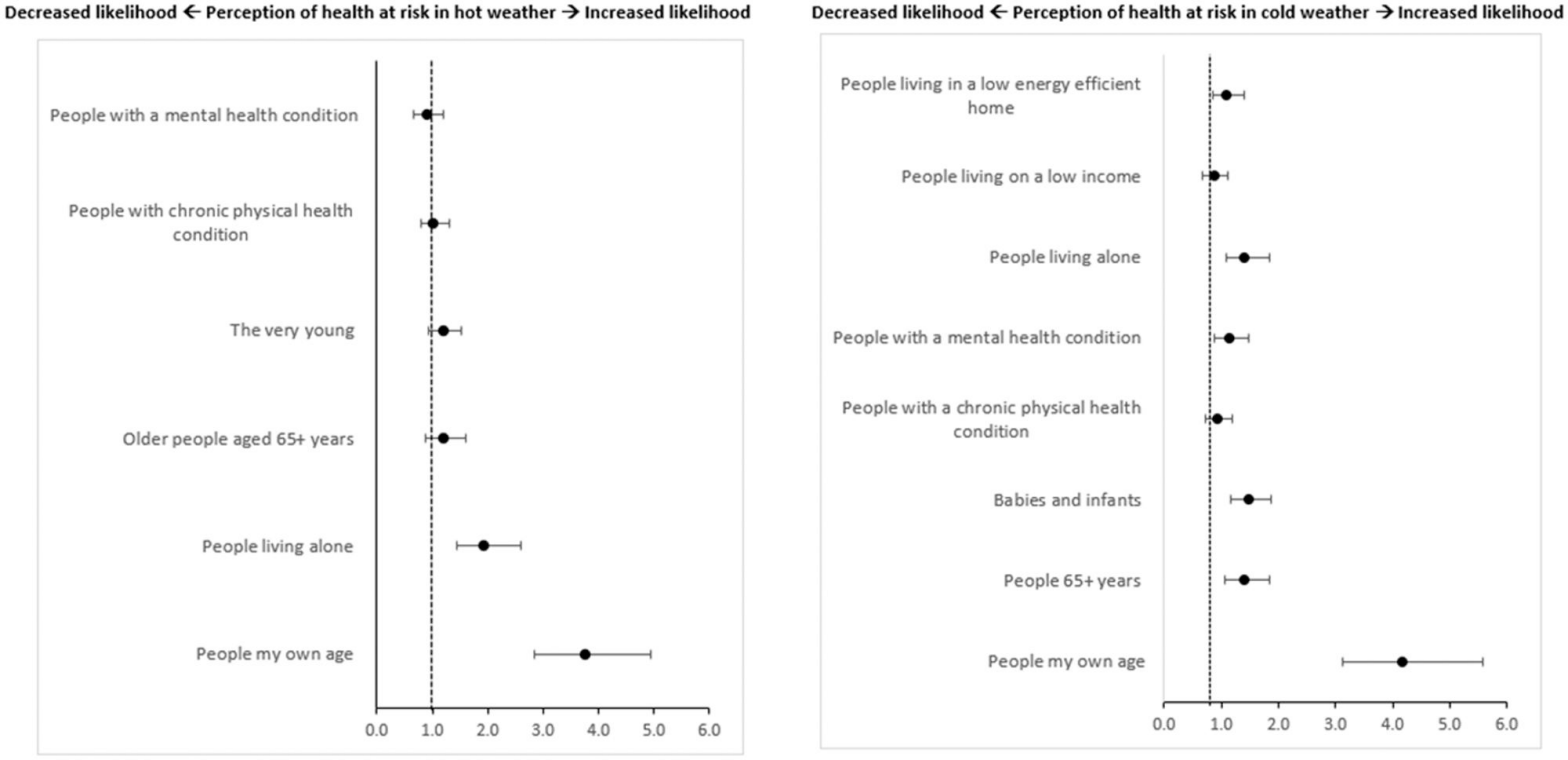
Analysis 2—binomial logistic regression model plot showing perception of other vulnerable groups risk in hot/cold weather as predictors of OAs (aged 65+ years) perception of risk to health from hot (left) and cold (right) weather. Figures show odds ratios. Adjusted for age, sex, education, income, home tenancy, and region.

A higher percentage of OA who perceived their own health as at risk in hot weather also identified different vulnerable groups as at an increased risk (Supplementary material S6). Figure 2 presents the association between OA perception of other vulnerable groups health risk as predictors of personal risk perception in high/low temperatures. In both survey results, OA that identified people of the same age [heat: OR 3.75, 95% CI (2.85–4.95); cold: OR 4.17, 95% (3.12–5.58)] and people living alone [heat: OR 1.94, 95% CI (1.45–2.59); cold: OR 1.41, 95% CI (1.08–1.84)] as at an increased risk were significantly more likely to perceive their own health as at risk. Additionally, OA perceiving others aged 65+ as at risk in cold weather was significantly associated with OA perceiving their own health to be at risk [OR 1.4, 95% CI (1.06–1.84)]. Identifying babies and infants [OR 1.48, 95% CI (1.16–1.87)] as at an increased risk in cold weather was a significant predictor of OA perceiving their own health as at risk.

### Behavioural responses as predictor of OA risk perception

There were no notable differences in the proportion of O reporting at least one action to reduce the impact of cold (89%) and the impact of heat (90%). More than half of OA reported the following heat-related behaviours: drinking more fluids (84%), opening windows at night/cooler parts of the day (61%), wearing loose clothing and/or a hat (59%), finding somewhere that felt cool (53%), and keeping curtains closed on windows exposure to direct sunlight (50%). Over half of OA reported the following cold-related responses: layering clothing (80%), heating home >18°C (77%), having their boiler checked by an engineer (61%), heating rooms most occupied (59%), keeping bedroom window closed at night (59%), checking the forecast and planning ahead (53%), and drinking warm drinks (58%).

**Figure 3.**
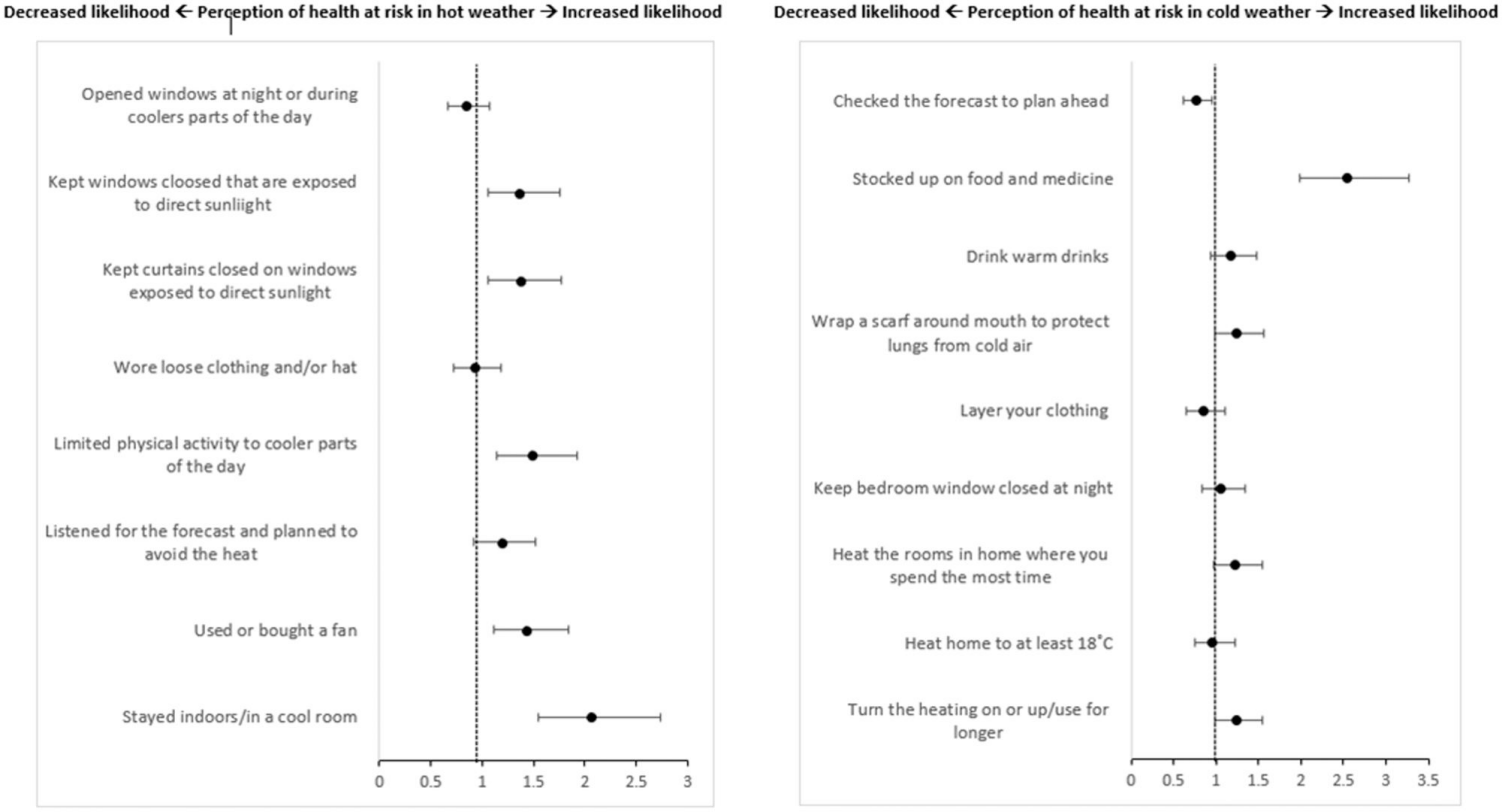
Analysis 3—binomial logistic regression model plot showing temperature-related behaviours as predictors of OA (aged 65+ years) perception of risk to health from hot (left) and cold (right) weather. Figures show odds ratios. Adjusted for age, sex, education, income, home tenancy, and region.

Some health-protective responses and behaviours to hot and cold weather were associated with OA perception of risk (Fig. 3). Reporting using or buying a fan during hot weather was 43% more likely to agree their health to be at risk in hot weather [OR 1.43, 95% (1.11–1.84)]. OA who reported staying indoors [OR 2.06, 95% CI (1.55–2.74)] or limited physical activity to cooler parts of the day [OR 1.48, 95% CI (1.14–1.92)] were significantly more likely to perceive their health as at risk in hot weather. Keeping curtains or windows closed exposed to direct sunlight were statistically significant predictors for OA being more likely to agree their health is at risk in hot weather [OR 1.37, 95% CI (0.31–1.77)]. In cold weather, OA who stocked up on food and medicine [OR 2.54, 95% CI (1.98–3.27)] were more likely to perceive their health to be at risk. Finally, OA who checked the forecast and planned ahead were 23% less likely to agree that their health is at risk during cold weather [95% CI (0.62–0.95)].

## Discussion

Our findings align with previous evidence that less than half of OA in England consider their health to be at an increased risk in hot or cold weather events [5, 29]. In the evaluation of the HWP, fewer OA reported their health as at risk in hot weather compared to this more recent survey, possibly due to an increase in frequency and intensity of heatwaves [30]. Previous research indicates the following factors to influence HRP in OA: conceptualization of age identity, lived experiences, stoicism, or misconceptions of public health messaging [5, 31]. Furthermore, we see in this study that demographic determinants and some behavioural responses are linked with perceptions of risk in hot and cold weather which highlights the complexity in understanding what predicts OAs vulnerability and perception of their own risks. Future research should ascertain whether changing frequency and intensity of extreme temperatures has altered OA health risk awareness to establish whether this aids or undermines individual action and response.

This study identified demographic factors that influence OA HRP in extreme weather. Those with higher education, income, or home ownership were less likely to perceive health risks during cold weather. Fewer barriers, greater resources, and knowledge of cold impacts can reduce health risks, such as affording heating for longer or living in better-quality housing [18]. In hot weather, OAs in the South of England were 19% less likely to see themselves at risk compared to those in the Midlands or North. This may be due to Southern residents being more accustomed to warmer temperatures, leading to underestimation of risks. Differences in HRP may result from various behaviours during peak sun hours or fewer heat events in the North [32]. Additionally, as OAs in the South frequently face heat risks, they might adopt more protective behaviours, perceiving lower health risks. Existing literature indicates that heat-related mortality is higher in southern regions under climate change while northern regions face greater cold-related mortality [4]. Although age and gender differences were not found in this analysis, previous studies showed women over 75 are more likely to recognize heat-related health risks [14, 30]. Understanding demographic differences in HRP during extreme weather is crucial for effective risk communication and response uptake. For instance, the income and education relationship identified suggests that infrastructure improvements for heating and insulation may not be feasible for lower-income groups, while more affluent individuals may already be implementing such measures.

This analysis revealed differences in how OA recognize health risks for vulnerable groups during extreme weather. OA who identified ‘people of the same age’ as at risk were three times more likely to see themselves as vulnerable in hot or cold climates. However, when the term was changed to ‘older people aged 65+’, this association was significant only in the cold weather survey. OA may respond better to guidance that reflects peers they relate to rather than focusing on age alone. A disconnect exists among some OA regarding the link between advanced ageing and increased weather risks, indicating a need to explore self-identification and vulnerability characteristics as risk perception predictors [33, 34]. OA who recognized health risks for individuals living alone correlated with perceiving their health as at risk in both surveys. Public health professionals should understand that OA might see living alone, rather than age, as the main vulnerability factor. Those living alone may face social isolation and limited healthcare access, increasing their HRP during adverse weather [35]. Disparities in OA understanding how comorbidities may worsen health risks in extreme temperatures could stem from a failure to recognize their vulnerability, hindering action, and compliance with recommendations. Defining vulnerability among OA is crucial for the effective prioritization of care services, minimizing adverse effects of temperature extremes.

Variation in uptake of recommended acute health-protective behaviours among OA was noted, with limited reporting of regularly applying SPF or avoiding alcohol during hot weather [24]. Conversely, the most common heat-related behaviours in this study (e.g. drinking cool fluids, opening windows at night) were poorly adopted by OA outlined in the Heatwave Plan for England [5, 24, 25]. This variation may be linked to OA motivation, which may not prioritize health protection. Evidence suggests that a lower perception of risk can stem from a cultural desire to embrace hot weather [25, 36]. However, there are indications of increased risk awareness among OA in places like Hong Kong [31]. In this study, heat-related behaviours associated with OA perception of their health risks in hot weather included altering daily activity and modifying the home environment. Previous research indicates these behaviours are typically motivated by alleviating discomfort from heat exposure and reducing UV exposure [22, 25]. Common home modifications included closing windows and curtains, using fans, and creating cool rooms. In contrast, altering indoor temperatures during cold weather was not related to risk perception. Differences in temperature-related behaviours may arise from habitual responses, such as heating the home in winter versus cooling it in summer . OA who checked forecasts for cold weather were less likely to perceive health risks, potentially viewing health protection behaviours as common sense. Those stocking up on food and medicine due to cold weather were 2.5 times more likely to perceive their health as at risk. OA engaging in health-protective behaviours tend to be more health-conscious or in poorer health. These findings are crucial for public health messaging during heat or cold events, emphasizing immediate actions for OA to protect their health within a short timeframe (e.g. 48 h before the event).

### Strengths and policy implications

This is a comprehensive study of HRP in hot and cold weather amongst OA conducted in person and was nationally representative in England. The results from this study show that perception of the risks of hot and cold weather remain low, and protective actions are varied, even among high risk populations such as OA. The findings from this analysis are important for public health agencies for implementing successful national and regional risk communication strategies, as well as guiding future research as called for following the Environmental Audit Committee inquiry published in early 2024 [37] (see Supplementary material S6) . Additionally, including survey data from both a hot weather and cold weather event allowed for a comparison of the differences in OA risk perception in differing temperatures and the predicting factors which may have influence.

### Limitations

The survey did not capture respondents’ health status, so we could not compare risk perception between health profiles. However, we examined age range (65–74 versus 75+) as a proxy for frailty and found no differences in risk perception. July 2019 had three heatwaves and above-average rainfall which may have affected participants’ recollection of heat-related behaviours. The cold weather survey followed a mild winter, possibly explaining low reporting of behaviours. The sample of OA was not representative for ethnicity, with over 96% being White British. While ethnicity has been cited as a possible risk modifier, we could not assess this due to the small sample proportion of non-white British people. Urban/rural information was not captured, which is important for understanding HRP in cold weather (e.g. healthcare access disruptions from snow or ice).

## Conclusion

This study found that most OA do not perceive their health as at risk during hot or cold weather. The analysis identified demographic predictors of HRP for OA, influenced by factors such as region for hot weather and income, education, and home ownership for cold weather. It also revealed how OA views on vulnerable groups vary based on the language used to self-identify age and vulnerability. Both surveys indicated variability in the uptake of health-protective behaviours, with some behaviours linked to HRP. Key barriers to recognizing health risks among OA include self-identification of vulnerability, stoicism, terminology, awareness of health risks, and cultural norms. These findings are vital for public health agencies to tailor temperature-related communication strategies and health messaging for OA. Future research should further explore barriers to risk perception and the adoption of health-protective behaviours among OA.

## Supplementary Material

ckae153_Supplementary_Data

## Data Availability

The data underlying this article were accessed from IPSOS Mori (https://www.ipsos.com/en-uk). The derived data generated in this research will be shared on reasonable request to the corresponding author. Key points Less than half of older adults (OA) in this study perceive hot or cold weather as a risk to their health. Regional differences in health risk perception in OA in hot weather and demographic predictors of cold weather being a risk to health were income, education, and home ownership. Key temperature-related behaviours associated with perceiving health as at risk in hot or cold weather were related to home modifications and adjusting daily activity. Persistent barriers to recognizing health risk in OA include self-identification of vulnerability, stoicism, terminology, awareness of health risks, and cultural norms. Importance of these findings for aiding public health agencies to target and modify temperature-related risk communication strategies and health messaging to engage OA.
