# Supplementary material for: Health perceptions of adverse weather in older adults in England: analysis of 2019/20 survey data
Source: Eur J Public Health. 2024 Oct 24;34(6):1192–8. doi: 10.1093/eurpub/ckae153 (PMC11631402; doi:10.1093/eurpub/ckae153)
Supplement: ckae153_Supplementary_Data [file ckae153_supplementary_data.docx]

**Supplementary material**

S1 – In person survey questions

IPSOS - Extreme weather heat questionnaire

**Q1 Which of the following best describes how you feel about hot weather in general?**

1. I love it
2. I like it
3. I don’t mind it
4. I dislike it
5. I hate it
6. Don’t know

**Q2 To what extent do you agree or disagree with the following statement? Hot weather is a risk to my health**

1. Strongly agree
2. Agree
3. Neither agree nor disagree
4. Disagree
5. Strongly disagree

**Q3 Hot weather is a risk to the health of which of the following groups of people, if any?**

1. People my own age
2. Older people (65 years or over)
3. The very young
4. People with a long-standing physical health condition
5. People with a mental health condition
6. People living on their own
7. None of the above
8. Don’t know

**Q4 Most parts of England experienced hot weather in the last few weeks. Were you in England during that time?**

1. Yes – in England during some or all of that time
2. No – out of England during that time

**Q5 During the recent hot weather, did you ever feel uncomfortably hot?**

1. Yes
2. No
3. Can’t remember

**Q6 What, if anything, did you do to reduce potential harm to your health from the recent hot weather?** ADD IF NECESSARY: **And what else did you do?** OPEN ENDED

**Q7 You say you didn’t take any action to reduce the potential harm to your health from the recent hot weather. Why was this?** OPEN ENDED

**Q8 During the recent hot weather which of the following actions, if any, did you take to reduce potential harm to your health from the hot weather?**

1. Drank more fluids
2. Listened out for the forecast and then planned ahead to avoid the heat
3. Found somewhere that felt cool
4. Limited strenuous physical activity to the cooler parts of the day
5. Wore loose clothing and/or a hat
6. Used an electric fan
7. Kept curtains closed on windows exposed to direct sunlight during the day
8. Kept windows closed that are exposed to direct sunlight during the day
9. Opened windows at night or during the cooler parts of the day
10. Avoided alcohol
11. Sought professional health advice
12. None of the above NOT ON SHOWSCREEN (SINGLE CODE)
13. Can’t remember NOT ON SHOWSCREEN (SINGLE CODE)

**Q9 You didn’t take some of the actions we’ve just mentioned to reduce potential harm to your health from the recent hot weather. Why was this?** PROMPT IF NECESSARY WITH THE ACTIONS NOT TAKEN

1. Didn’t think there was a need
2. It wasn’t possible
3. My health wasn’t at risk
4. Didn’t think this would be relevant to me
5. Didn’t think it would make a difference
6. It didn’t occur to me
7. It wasn’t hot enough
8. Couldn’t afford it
9. Other (please specify)
10. Don’t know NOT ON SHOWSCREEN (SINGLE CODE)

**Q10 During the recent hot weather which of the following actions, if any, did you take to reduce potential harm to the health of others you know who are very young, or who are older, or who have a long-standing health condition?**

1. Checked to see if the heat was affecting them
2. Ensured they drank more fluids
3. Listened out for the forecast and planned ahead to help them to avoid the heat
4. Ensured that they were somewhere cool
5. Limited their strenuous physical activity to the cooler parts of the day
6. Ensured that they wore loose clothing and/or a hat
7. Used an electric fan
8. Kept curtains closed on windows exposed to direct sunlight during the day
9. Kept windows closed that are exposed to direct sunlight during the day
10. Opened windows at night or in the cooler parts of the day
11. Avoided providing alcohol
12. Sought professional health advice
13. None of the above
14. Can’t remember

**Q11 How did you know what action to take to reduce the potential harm to your health, and the health of others, of hot weather?**

1. I had heard/seen advice and publicity on what to do
2. I always do this
3. I was advised to by a professional
4. Other (please specify)
5. Don’t know

**Q12 At any time during this summer, did you experience any of the following as a result of hot weather or heat?**

1. Difficulty keeping cool/ feeling too hot
2. Difficulty sleeping
3. Physical symptoms (e.g. headaches, dehydration, sunburn)
4. A need to seek help from health services or other support services
5. Other (please specify)
6. Can’t remember

**Q13 Listed below are some health symptoms. Do you think the following symptoms can occur as a result of heat?**

1. Fever
2. Heart problems (e.g. heart attack)
3. Lung problems (e.g. difficulty breathing)
4. Diarrhoea
5. Death

IPSOS – Extreme weather cold questionnaire

**Q1 To what extent do you agree or disagree with the following statement? Cold weather is a risk to my health**

1. Strongly agree
2. Agree
3. Neither agree nor disagree
4. Disagree
5. Strongly disagree

**Q2 Cold weather is a risk to the health of which of the following groups of people, if any?**

1. People my own age
2. People 65 years or over
3. Babies and infants
4. Teenagers
5. People with a long-standing physical health condition
6. People with a mental health condition
7. People living on their own
8. People living on a low income
9. People living in a home that is not very energy efficient
10. None of the above
11. Don’t know

**Q3 When there is a spell of particularly cold weather (such as when there is frost or temperatures drop), what, if anything, do you typically do to reduce potential harm to your health from the cold weather?** ADD IF NECESSARY: **And what else do you typically do?** OPEN ENDED

**Q4 You say you don’t typically take any action to reduce the potential harm to your health during cold weather. Why is this?** OPEN ENDED

**Q5 Still thinking about particularly cold spells of weather (such as when there is frost or temperatures drop), which of the following, if any, do you typically do to reduce potential harm to your health from the cold weather?**

1. Heat your home to at least 18 degrees
2. Heat the rooms in your home where you spend most time so they are warmer than other rooms
3. Check the forecast so you can plan ahead
4. Keep your bedroom window closed at night
5. Layer your clothing (whether indoors or outside)
6. Wrap a scarf around your mouth to protect your lungs from cold air when outdoors
7. Move around at least once an hour to keep warm
8. Drink warm drinks
9. None of the above

**Q6 You said you do not typically do some [any] of the actions we’ve just mentioned to reduce potential harm to your health from cold weather. Why is this?**

1. I didn’t know these actions were recommended
2. It’s too difficult physically
3. It’s too stressful
4. I just put up with the cold
5. I don’t want to spend my money on this
6. I don’t think there is a need
7. My health isn’t at risk
8. I don’t think it would make a difference
9. It doesn’t occur to me
10. It hasn’t been cold enough
11. It’s too expensive
12. Other (please specify)
13. Don’t know

**Q7 And which of the following, if any, have you ever done to reduce potential harm to your health from cold weather?**

1. Sought financial support (e.g. to help with heating bills)
2. Sought treatment for minor winter ailments (e.g. a cold or sore throat)
3. Sought advice (e.g. from family members, a health or social care professional, or your local council) about how to keep warm
4. Had heating and cooking appliances checked by a Gas Safe registered engineer
5. Had your boiler checked by a Gas Safe registered engineer
6. Contacted water and power suppliers to see if you could be on the Priority Services Register
7. Stocked up on food and medicine to avoid going outside
8. Checked the forecast to plan ahead
9. None of the above
10. Can’t remember

**S2 Temperature related symptoms**

Older adults reported experiencing at least one symptom during hot (76%) or cold weather (40%). The most commonly reported heat-related symptoms included experiencing difficulty keeping cool and feeling too hot (40%), having difficulty sleeping (52%) and physical symptoms such as a headache, dehydration or sunburn (11%). Older adults reported fewer cold-related symptoms (60% reported no symptoms following 2019 winter).

**S3 Full Ethics statement**

“This project was reviewed by the internal IPSOS MORI Ethics Group, but it was not submitted externally for ethics approval which is standard practice in market research where the research topic is not considered sensitive and the participants are not considered vulnerable or unable to give informed consent. It was also judged that there would be no potential disclosure of harm through the survey. The data received by the authors in this study was anonymised and individuals are not identifiable.”

**S4 – Summary table: Older adults health risk perception in hot and cold weather (Analyses 1 – 3)**

| **Predictor** | **Heat survey respondents**  **(*n = 452*)** | **Adj OR**  **[95% CI]** | **Cold survey respondents**  **(*n = 461*)** | **Adj OR**  **[95% CI]** |
| --- | --- | --- | --- | --- |
| **Age**  1 = 75+  0 = 65 - 74 | *N = 211 (47%)* | 0.96 [0.72 – 1.28] | *N = 187 (41%)* | 0.9 [0.69 – 1.19] |
| **Sex**  1 = Female  0 = Male | *N = 233 (52%)* | 1.21 [0.98 – 1.48] | *N = 208 (44%)* | 1.02 [0.84 – 1.25] |
| **Education**  1 = Degree level or higher  0 = Below degree level/no education | *N = 177 (39%)* | 0.89 [0.72 – 1.1] | *N = 147 (31%)* | 0.71 [0.56 – 0.89] |
| **Household annual income**  1 = >£25,000  0 = <£25,000 | *N = 123 (27%)* | 0.84 [0.67 – 1.05] | *N = 120 (25%)* | 0.75 [0.6 – 0.94] |
| **Tenancy**  1 = Home owned  0 = Home rented | *N = 380 (84%)* | 1.11 [0.89 – 1.39] | *N = 375 (79%)* | 0.64 [0.51 – 0.79] |
| **Region ^a^**  1 = South England (heat survey)  0 = Midlands or North | *N = 237 (52%)* | 0.81 [0.66 – 0.99] | *N = 213 (46%)* | 1.22 [0.99 – 1.49] |

*Table S4: Analysis 1 Binomial logistic regression model between participant (older adults aged 65+ years) health risk perception in high/low temperatures and possible predictors. ^a^ Regional analysis of cold survey participants variable coding: 1 = North or Midlands, 0 = South of England*

| **Predictor ^a^** | **Heat survey respondents**  **(*n = 452*)** | **Adj OR**  **[95% CI]** | **Cold survey respondents**  **(*n = 461*)** | **Adj OR**  **[95% CI]** |
| --- | --- | --- | --- | --- |
| People own age at risk | *N = 197 (44%)* | 3.75 [2.85 – 4.95] | *N = 201 (44%)* | 4.17 [3.12 – 5.58] |
| Older people (>65) at risk | *N = 334 (74%)* | 1.2 [0.89 – 1.61] | *N = 291 (63%)* | 1.4 [1.06 – 1.84] |
| The very young | *N = 272 (60%)* | 1.19 [0.92 – 1.52] | *N = 235 (51%)* | 1.48 [1.16 – 1.87] |
| People with long-standing physical condition | *N = 272 (60%)* | 1.02 [0.79 – 1.31] | *N = 303 (66%)* | 0.92 [0.72 – 1.18] |
| People with mental health condition | *N = 141 (31%)* | 0.9 [0.67 – 1.2] | *N = 176 (38%)* | 1.14 [0.87 – 1.48] |
| People living alone | *N = 143 (32%)* | 1.94 [1.45 – 2.59] | *N = 209 (45%)* | 1.41 [1.08 – 1.84] |
| People living on a low income | *-* | - | *N = 277 (60%)* | 0.87 [0.67 – 1.12] |
| People living in a home that is not very energy efficient | *-* | - | *N = 201 (44%)* | 1.08 [0.84 – 1.4] |

*Table S4: Analysis 2 Binomial logistic regression model between participant (older adults aged 65+ years) health risk perception during hot or cold weather and potential predictors.* Adjusted for age, sex, education, income, home tenancy and region. ^a^  1 = agree sub-group as at risk during hot or cold weather, 0 = do not agree sub-group as at risk during hot or cold weather.

| **Heat-related behaviour ^a^** | **Number (%) of respondents**  *(n = 452)* | | **Adj OR [95% CI]** | |  |
| --- | --- | --- | --- | --- | --- |
| Used or bought a fan | *n = 99 (22%)* | | 1.43 [1.11 – 1.84] | |  |
| Stayed indoors | *n = 103 (23%)* | | 2.06 [1.55 – 2.74] | |  |
| Listened to the forecast and planned ahead to avoid the heat | *n = 179 (40%)* | | 1.19 [0.92 – 1.52] | |  |
| Limited strenuous physical activity to cooler parts of the day | *n = 193 (43%)* | | 1.48 [1.14 – 1.92] | |  |
| Kept curtains closed on windows exposed to direct sunlight | *n = 225 (50%)* | | 1.37 [1.06 – 1.77] | |  |
| Kept windows closed that are exposed to direct sunlight | *n = 143 (32%)* | | 1.36 [1.05 – 1.76] | |  |
| Wore loose clothing and/or a hat | *n = 266 (59%)* | | 0.92 [0.72 – 1.18] | |  |
| Opened windows at night or during cooler parts of the day | *n = 275 (61%)* | | 0.84 [0.66 – 1.07] | |  |
| **Cold-related behaviour ^a^** | | **Number (%) of respondents**  *(n = 461)* | | *Adj OR [95% CI]* | |
| Stocked up on food and medicine | | *n = 139 (30%)* | 2.54 [1.98 – 3.27] | | |
| Drank warm drinks | | *n = 267 (58%)* | 1.17 [0.93 – 1.48] | | |
| Keep bedroom window closed at night | | *n = 272 (59%)* | 1.06 [0.84 – 1.34] | | |
| Checked forecast to plan ahead | | *n =250 (54%)* | 0.77 [0.62 – 0.95] | | |
| Turn the heating on or up / use for longer | | *n = 184 (40%)* | 1.24 [0.99 – 1.54] | | |
| Heat home to at least 18˚C | | *n = 357 (77%)* | 0.96 [0.76 – 1.22] | | |
| Heat the rooms in home where you spend most time | | *n = 276 (60%)* | 1.23 [0.98 – 1.54] | | |
| Layer clothing | | *n = 371 (80%)* | 0.85 [0.65 – 1.1] | | |
| Wrap a scarf around mouth to protect lungs from cold air | | *n = 155 (34%)* | 1.24 [0.99 – 1.57] | | |

*Table S4: Analysis 3 Binomial logistic regression model between participant (older adults aged 65+ years) health risk perception from high/low temperatures and temperature-related measures or behaviours. Adjusted for age, sex, income, education, home tenancy and region. ^a^  1 = reported behaviour during cold or hot weather, 0 = did not report behaviour during hot or cold weather.*

**S5 - Regional differences in older adults risk perception in hot and cold weather**

*Figure S5: Percentage of older adults who perceive their health to be at risk in hot/cold weather by region in England. Hot weather survey n = 452, cold weather survey n = 461.*

**S6 – Percentage of older adults risk perception of other vulnerable groups during hot or cold weather**

In both surveys, a larger proportion of older adults who disagreed that their own health is at risk also disagreed that ‘people aged 65 years and over’ were at risk, however, less disagreed with ‘people of the same age’ being at risk. It appears that there is varying consistency regarding older adults’ awareness of their own health risk and risk perception of other vulnerable groups in relation to high and low temperatures.

*Figure S4: Percentage of older adult respondents who perceive other groups health to be at risk in high/low temperatures by their personal perception of the risk to their own health . (Heat survey n = 452, cold survey n = 461)*

**S6 – Recommendations for policy, public health and future research**

**Box 1: *Recommendations for policy, public health agencies and future research***

Risk communication and public health messaging**:**

- Public engagement to focus on emphasising who is most at risk through targeted messaging for OA through accessible channels e.g. weather forecast, traditional media, GP surgeries, awareness of efficacy of actions, promoting less successfully adopted behaviours and motivation strategies
- Consider the importance of demographics, region, terminology and language to minimise risk as individual identification of social vulnerability differs amongst OA
- Individual perspectives and social norms are likely to play an important role in perceptions of risk in hot and cold weather e.g. attitudes, stoicism or ambivalence towards health risks to adverse weather
- Continue to prioritise health and care services, particularly for OA living alone and those with co-morbidities

Future research:

- Explore solutions and strategies for increasing understanding and public awareness around the risks from heat and cold
- Identify the relationship between the changes in frequency and intensity of extreme temperatures on public health risk perception
- More information on how to encourage people to take specific actions that are less commonly adopted but of public health significance e.g. seeking treatment, avoiding alcohol during hot weather
- Utilise insights from this research to develop series of messages and communication material to trial and evaluate using robust behavioural science methods
